# Latency Period Among Asbestosis Cases in South Korea by Demographic and Asbestos Exposure Characteristics

**DOI:** 10.3390/toxics13090775

**Published:** 2025-09-13

**Authors:** Won Young So, Min-Sung Kang, Young Hwangbo, Mee-Ri Lee

**Affiliations:** 1 Asbestos Environmental Health Center, Soonchunhyang University Cheonan Hospital, Soonchunhyang 6-gil 31, Dongnam-gu, Cheonan-si 31151, Republic of Korea; sch83sch@naver.com (W.Y.S.); kms83korea03@hanmail.net (M.-S.K.); 2 Division of Medical Science, Soonchunhyang University, 22, Soonchunhyang-ro, Sinchang-myeon, Asan-si 31538, Republic of Korea; 3 Department of Preventive Medicine, College of Medicine, Soonchunhyang University, Soonchunhyang 6-gil 31, Dongnam-gu, Cheonan-si 31151, Republic of Korea; hbyoung@sch.ac.kr

**Keywords:** asbestos, asbestosis, latency period, environmental asbestos exposure, occupational asbestos exposure

## Abstract

Although asbestos use has been banned in many countries, including South Korea, the long latency period of asbestos-related diseases remains a serious public health concern. We conducted a nationwide, registry-based retrospective study to estimate the latency period of asbestosis and identify its determinants. We analyzed exposure history and demographics for 1110 asbestosis cases collected by the Ministry of Environment and the Environmental Health Center for Asbestos in Korea between 2009 and 2021. Mean latency was 45.3 years for asbestosis Grade 1 and 46.3 years for Grade 2. Patients with occupational exposure had shorter latency than those with environmental exposure: 44.4 vs. 46.0 years in Grade 1 (*p* = 0.010) and 45.0 vs. 47.0 years in Grade 2 (*p* < 0.001). Within occupations, production-type work showed the shortest latency; within environmental exposure, residence near asbestos-related industries showed the shortest latency, whereas residence near asbestos mines showed the longest. Longer exposure duration (occupational) was associated with shorter latency, and greater residential distance from the source (environmental) with longer latency. Priorities for further investigation include differences by asbestos fiber type and exposure intensity/modality, to inform strengthened occupational health monitoring and targeted surveillance for residents near industrial sources and legacy mines.

## 1. Introduction

Asbestosis is a chronic interstitial lung disease caused by prolonged inhalation of asbestos fibers and is characterized by progressive pulmonary fibrosis [1,2]. Although asbestos use has been banned in many countries, including Korea, the long latency period of asbestos-related diseases continues to pose a serious public health challenge. In Korea, amphibole asbestos types were banned in 2003 and chrysotile in 2009; subsequent regulations have strengthened control over legacy sources, providing important policy context for disease surveillance. However, individuals exposed to asbestos decades ago remain at risk of developing asbestosis, making it a persistent concern despite regulatory efforts [3]. To address these issues, the Ministry of Environment of Korea enacted the Asbestos Injury Relief Act in 2011 and designated Environmental Health Centers for Asbestos to operate a health surveillance system aimed at identifying asbestos victims [4].

Understanding the latency period of asbestos-related diseases is essential for effective disease surveillance, prediction, and prevention. The latency period, which often spans several decades, significantly influences the timing of symptom onset, diagnosis, and public health response [5,6]. A precise understanding of latency can enhance the accuracy of forecasting future disease burden, especially in populations exposed in the past, and help identify at-risk groups who may otherwise be overlooked [5,6,7]. Furthermore, insights into latency are crucial for designing targeted early detection programs, establishing timely compensation mechanisms, and developing policies to protect vulnerable populations from ongoing or legacy asbestos exposure [7,8,9]. Investigating the determinants of latency also provides a foundation for refining exposure assessments and risk stratification models used in epidemiological and policy research [10,11]. In addition, from a policy perspective, clarifying the latency period and its influencing factors can help optimize the national compensation mechanism under the Asbestos Injury Relief Act. For example, claim time criteria and eligibility windows can be calibrated to empirically supported latency ranges; presumptive thresholds can be differentiated by exposure modality (occupational vs. environmental) or intensity; and case review can be prioritized for groups with demonstrably shorter latency. Such evidence-based adjustments may reduce under-compensation of late-presenting cases, improve fairness in adjudication, and align surveillance and resource allocation with actual risk profiles.

In Korea, several studies have attempted to estimate the future burden of asbestos-related diseases using statistical approaches such as the age–period–cohort (APC) model or Poisson regression [12,13,14]. These models play a critical role in forecasting long-term disease trends and informing national health policy [15]. However, the reliability of such projections depends on the accurate specification of the latency period for each disease. Reported latency periods for mesothelioma, lung cancer, and asbestosis vary widely across populations and study designs, reflecting differences in exposure characteristics, occupational environments, and individual-level risk factors [16,17]. Over the past 5–10 years, international studies have further clarified determinants of latency, highlighting the roles of age at first exposure, exposure intensity and modality (occupational vs. environmental), cumulative exposure, and fiber type; however, most of this work focuses on malignant outcomes rather than asbestosis [5,6,18]. Therefore, to enhance precision, it is essential to conduct research that utilizes large-scale, nationally representative datasets, particularly those collected through government-led health surveillance systems.

Despite its clinical and policy relevance, few studies have examined the latency period of asbestosis [7,15]. Existing research has predominantly focused on mesothelioma or lung cancer, with limited attention to asbestosis [5]. However, latency in asbestosis is known to vary depending on exposure intensity and duration, fiber type, occupational setting, smoking status, and underlying pulmonary conditions [7,18,19]. Understanding these factors is crucial for improving disease forecasting, optimizing health surveillance programs, and refining compensation schemes under the Asbestos Injury Relief Act. To our knowledge, no dedicated, nationwide study has characterized the latency of asbestosis in South Korea, underscoring the need for comprehensive, registry-based analyses.

Therefore, this study aims to investigate the latency period of asbestosis in Korea and identify key determinants affecting its duration. By analyzing national-level data, this research seeks to contribute to a more accurate understanding of disease progression in asbestos-exposed populations and to support evidence-based public health interventions and policymaking.

## 2. Materials and Methods

### 2.1. Data Source and Study Population

In this study, we utilized data on patients with asbestosis that were collected by the Ministry of Environment and Soonchunhyang University Cheonan Hospital, the Environmental Health Center for Asbestos in Korea, between 2009 and 2021. Under the Asbestos Damage Relief Act, the Ministry of Environment gathers information on individuals affected by occupational or environmental asbestos exposure through three pathways. First, individuals seeking compensation for asbestos-related diseases submit details regarding their asbestos exposure and medical history to local governments. Second, as part of mandated health screenings under the National Health Insurance Act, medical institutions are required to notify the Ministry if signs suggestive of asbestos-related damage are detected. Third, since 2009, the Environmental Health Center has identified victims of environmental asbestos exposure. The Center defined areas within a 2 km radius of asbestos sources, such as mines, manufacturing facilities, shipyards, and asbestos-containing buildings, as presumed exposure zones. It has conducted health surveys and epidemiological studies targeting individuals who have resided in these areas for over a decade.

**Figure 1:**
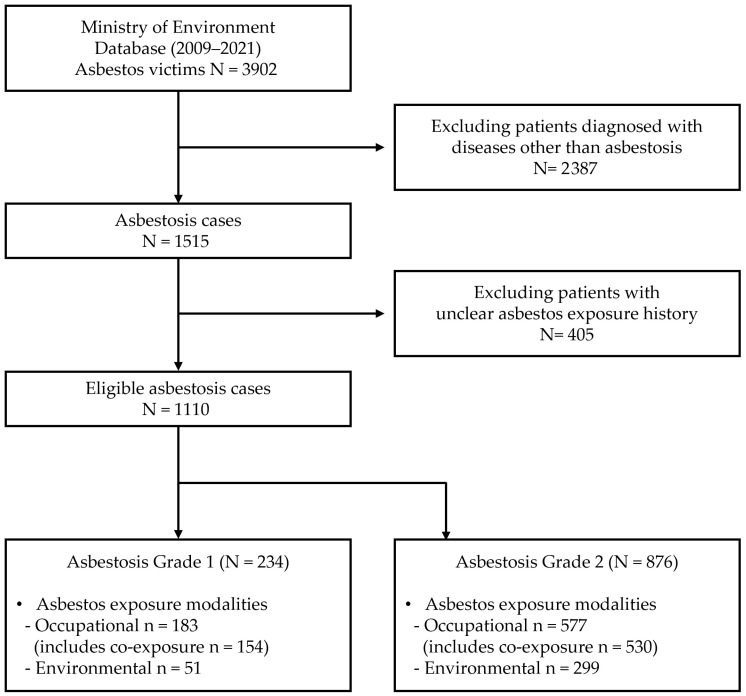
Flow diagram describing the inclusion and exclusion process of study participants.

The Korea Environmental Industry and Technology Institute, under the Ministry of Environment, assessed the causal relationship between asbestos exposure and disease occurrence based on the collected exposure histories and clinical data. Through this system, we obtained data on 3902 individuals officially recognized as asbestos victims by 2021. We excluded individuals with diagnoses other than asbestosis (*n* = 2387) and those with unclear exposure histories (*n* = 405). A total of 1110 cases were included in the final analysis (Figure 1).

The institutional review board of Soonchunhyang University Cheonan Hospital approved the collection and utilization of data for this study (2009-04-001).

### 2.2. Asbestos Exposure Assessment

Information on lifetime asbestos exposure was gathered by trained researchers at the Environmental Health Center using structured questionnaires developed by the Ministry of Environment. Asbestos exposure was classified into occupational and environmental exposure based on the type of exposure.

Occupational asbestos exposure was defined as having engaged in work involving asbestos fiber contact for a minimum duration of one year. Detailed data were also collected, including workplace name, job type, length of employment, and age at first exposure. To reduce potential information bias during data collection, participants’ self-reported histories were cross-verified against at least one independent historical source, including company and business registries, operating permits, and local-government factory/mining licenses documenting years and locations of operation, government inspection reports, environmental/industrial-hygiene records, and historical aerial or cadastral maps. Occupational types were categorized into four groups: (1) construction or demolition of buildings containing asbestos-containing materials (e.g., insulation); (2) production of asbestos-containing products (e.g., cements, slates, and textiles); (3) maintenance and repair of asbestos-containing buildings or equipment; and (4) asbestos extraction, conveyance, and grinding.

Environmental asbestos exposure was defined as non-occupational exposure to airborne asbestos fibers from sources such as asbestos mines, industrial facilities, or storage sites, with a minimum of one year of residential exposure. The Environmental Health Center obtained detailed information on residential area, type of exposure source, proximity to the source, duration of residence, age at first exposure, and history of soil cultivation. To ensure accuracy, participants’ survey responses were verified using historical inventories of asbestos sources provided by the Ministry of Environment and resident-registration records.

Patients who experienced both occupational and environmental asbestos exposure were classified into the occupational exposure group, considering that occupational exposure levels are generally higher than environmental exposure levels. Specifically, among asbestosis cases, 154 patients in Group 1 and 530 patients in Group 2 reported both occupational and environmental exposure.

### 2.3. Latency Period

We defined latency periods as the duration between the initial asbestos exposure and the diagnosis of asbestosis, as determined from survey data. For occupationally exposed individuals, the time of initial exposure was defined as the age at which they began employment involving asbestos. In the case of environmentally exposed individuals, the first year of exposure was determined as either the year they started residing near the exposure sources or the year when those sources began operating.

### 2.4. Statistical Analysis

We conducted a univariate analysis to estimate the latency period stratified by participants’ age and gender. The mean and median values were reported as point estimates, while the standard deviation, range, and 5th and 95th percentiles were calculated to assess the variability in latency periods. Differences in the mean latency period across gender and age groups were evaluated using *t*-tests or analysis of variance (ANOVA).

To estimate adjusted latency periods for asbestosis, we employed analysis of covariance (ANCOVA). The analysis included the following covariates: gender (male, female), age (as a continuous variable), smoking status (never, past, or current smoker), asbestos exposure type (occupational or environmental), and age at initial exposure (continuous). These covariates were selected a priori based on prior literature and biological/epidemiological plausibility that they are associated with both exposure history and latency and may therefore act as confounders [5,7,18,19].

Furthermore, we examined the relationship between latency period and relevant factors using multiple linear regression analysis. In the occupational exposure model, regression coefficients were estimated for the exposure duration, age at initial exposure, and pack-years of smoking; these variables were included to capture dose and timing of exposure, which are established determinants of latency. In the environmental exposure model, we additionally included distance from the exposure source to the residence as a variable, reflecting proximity-related exposure intensity. In both models, age and gender were included as covariates.

## 3. Results

The general characteristics of the patients are presented in Table 1. In both asbestosis grade 1 and 2, the number of male patients was higher than that of female patients. The number of participants with occupational asbestos exposure exceeded those with environmental exposure, and the majority of cases were diagnosed between 2010 and 2019.

The latency periods for asbestosis Grade 1 and Grade 2 each followed a normal distribution (Table 2). The mean (standard deviation) latency period was 45.3 (14.4) years for asbestosis Grade 1 and 46.3 (14.9) years for Grade 2 (Table 2). Latency periods tended to increase with patient age, and a statistically significant difference by gender was observed in asbestosis Grade 2.

The adjusted mean latency periods are presented in Table 3. Among patients with asbestosis Grade 1, differences in latency by smoking status were not statistically significant, and the estimates were imprecise due to the small number of current smokers (*n* = 4); therefore, we did not compare this group directly with others. Patients with occupational asbestos exposure had significantly shorter latency periods (44.4 years) compared to those with environmental exposure (46.0 years) (*p* = 0.010). In addition, individuals who worked in production-type occupations or lived near asbestos industries exhibited the shortest latency periods (42.0 and 47.4 years, respectively); however, these differences were not statistically significant (*p* = 0.214 and 0.348, respectively). In the case of asbestosis Grade 2, similar to Grade 1, no significant differences in latency periods were observed according to smoking status, and estimates were imprecise given the small number of current smokers (*n* = 10). However, patients with occupational exposure had latency periods approximately two years shorter (45.0 years) than those with environmental exposure (47.0 years). In particular, among those with occupational exposure, patients who had worked in production-type occupations exhibited shorter latency periods (42.3 years) compared to those in other occupational categories (*p* < 0.001). Among those with environmental exposure, individuals exposed to asbestos from nearby asbestos industries had significantly shorter latency periods (50.4 years) than other groups (*p* = 0.001), whereas patients who had lived near asbestos mines showed the longest latency periods (53.8 years).

Table 4 presents the results of multiple regression analyses evaluating the association between latency period and each continuous variable. After adjusting for covariates, a significant negative linear association was observed in patients with occupational asbestos exposure, where longer exposure duration was associated with shorter latency periods. In contrast, among patients with environmental asbestos exposure, no significant association was found between exposure duration and latency period. However, a significant positive association was observed between the distance from the asbestos source to the residence and latency period, indicating that greater distances were associated with longer latency periods. In addition, age of first exposure showed a negative association with latency period. Regardless of asbestosis grade, for each one-year increase in age of first exposure, the latency period tended to decrease by approximately one year.

## 4. Discussion

The aim of this study was to estimate the latency period of asbestosis in South Korea and to identify the determinants influencing the latency period. A total of 1110 asbestosis cases collected by the Ministry of Environment and the Environmental Health Center for Asbestos were analyzed. In this study, the latency periods for asbestosis Grades 1 and 2 were 45.3 years and 46.3 years, respectively. Patients with occupational asbestos exposure had shorter latency periods compared to those with environmental exposure. Regarding occupational types, patients who had worked in industries producing asbestos-containing products exhibited shorter latency periods than those in other occupational groups. For environmental asbestos exposure, individuals residing near asbestos industries had the shortest latency periods, whereas those living near asbestos mines had the longest latency periods. In addition, among occupationally exposed participants, longer exposure durations were associated with shorter latency periods, while among environmentally exposed participants, greater distances from the exposure source were associated with longer latency periods.

Although recent studies focusing on the latency period of asbestosis are limited, Dominguez et al. investigated 642 patients in a Spanish outpatient cohort and reported a latency period of 48.13 ± 11.08 years [20], which is comparable to our mean latency estimates (Grade 1: 45.3 years; Grade 2: 46.3 years); however, several cohort- and methods-level differences could account for residual discrepancies. First, exposure intensity and modality likely differ across cohorts: our registry includes a substantial proportion of environmentally exposed residents in addition to workers, whereas outpatient cohorts are often predominantly occupational. Second, the distributions of age at first exposure and exposure duration can vary between settings; younger first exposure and longer duration are associated with shorter latency, so cross-cohort differences in these determinants would shift mean latency times. Third, diagnostic pathways and case ascertainment differ: in Korea, universal health coverage, regular national health screenings, and voluntary medical evaluations undertaken when applying under the Asbestos Injury Relief Act lead to more frequent and earlier detection than in many other countries. Consequently, the observed latency in our study may be slightly shorter than in settings with less systematic detection. In addition, various factors that may influence the latency period of asbestosis have been reported in multiple studies. These factors include age at first asbestos exposure [19,21], duration and intensity of asbestos exposure [19,21,22,23,24,25], individual susceptibility [21,22,23,24,25], type of asbestos fiber [21,23,24,25], and the presence of other respiratory irritants [22]. Among these, both age at first exposure and duration of exposure were confirmed as influential factors in our study as well. Reports on the effect of smoking on the latency period have been inconsistent. For example, a study by Baur et al. identified a significant impact of smoking on the latency of asbestosis [22], whereas Ferri et al. reported no significant association [19]. This discrepancy may be attributed to differences in how smoking exposure was assessed, specifically whether smoking was modeled categorically (never, former, current) versus as cumulative dose (pack-years), how former smokers were handled (e.g., time since quitting), and the timing/source of ascertainment (self-report at diagnosis versus historical records). In a study on the latency period of mesothelioma by Huh et al., current smokers exhibited latency periods 2–3 years shorter than those of never-smokers and former smokers [5]; however, when pack-years was modeled as a continuous variable, no linear association was observed, a pattern that is consistent with a potential non-linear or threshold relationship between smoking intensity and the latency period of asbestos-related diseases.

Asbestosis is known to develop following prolonged exposure to high concentrations of asbestos fibers, and is therefore commonly associated with occupational exposure [26]. However, emerging evidence suggests that even relatively low-level occupational exposure and environmental exposure, such as residing near asbestos mines, factories, or asbestos-containing buildings, can also pose significant risks for asbestosis, especially when fiber concentrations are elevated or exposure durations are prolonged. In Turkey, environmental exposure to tremolite asbestos in central Anatolia led to cases of diffuse interstitial pneumonia consistent with asbestosis, with one documented patient exhibiting 4250 asbestos bodies per milliliter in bronchoalveolar lavage fluid [27]. Similarly, a study in Da-yao, China, where crocidolite asbestos naturally occurs in surface soil, reported 16 cases of asbestosis among 2175 peasants in cross-sectional surveys [28]. Similarly, in our study, approximately 30% of asbestosis cases were attributable to environmental exposure. Moreover, the development of asbestosis has been shown to follow a dose–response pattern, with higher cumulative exposure linked to increased disease risk [29]. As observed in pneumoconiosis more broadly, there appears to be a threshold dose required to induce disease, influenced by both the intensity and duration of mineral inhalation [30]. Similarly, in our study, a significant negative correlation was observed between occupational exposure duration and latency period, and among environmentally exposed individuals, shorter distances from the exposure source to the residence were associated with shorter latency periods. These results suggest that the dose–response relationship between asbestos exposure and the development of asbestosis may also apply to the latency period.

We conducted a subgroup analysis of disease latency by further classifying participants based on their occupational types and sources of asbestos exposure. The findings showed that individuals involved in the production of asbestos-containing products and those residing near asbestos-related industries exhibited shorter latency periods compared to other groups. Conversely, those with occupational or environmental exposure related to asbestos mining demonstrated longer latency times. The toxicity of asbestos varies by fiber type, with chrysotile generally considered less harmful than crocidolite or amosite [16]. Notably, our registry did not include individual-level fiber-type detection (e.g., environmental or biological typing); therefore, the following interpretation is literature-based and should be considered inferential. Historical records on asbestos usage in Korea indicate that crocidolite and amosite were widely used in asbestos manufacturing plants [31]. According to Kim, approximately 40% of workers in asbestos factories were exposed to crocidolite [32]. In contrast, as chrysotile accounted for the majority of asbestos production in Korea [33], the exposure profiles of miners likely differed from those of factory workers. These differences suggest that exposure to more toxic asbestos types, such as crocidolite and amosite, may contribute to shorter latency periods. Because fiber-type monitoring data were unavailable for our cohort, alternative explanations—such as differences in exposure intensity (dust-generating processes, enclosure/ventilation) or co-exposures—cannot be excluded; future work linking geographic information with fiber-type measurements would strengthen this inference.

In our study, the latency period of asbestosis ranged widely from a minimum of 6 years to a maximum of 86 years, reflecting substantial inter-individual variation. Several plausible explanations may account for this variability. First, genetic factors play a critical role in individual susceptibility to asbestos-related diseases. Polymorphisms in glutathione S-transferase (GST) genes such as GSTM1 null [34], GSTT1 deletion [35], and high-activity GSTP1 genotypes have been associated with increased asbestosis risk [36], suggesting complex interactions in detoxification pathways. Variants in antioxidant enzymes like MnSOD and CAT have also been linked to elevated susceptibility [37,38]. These genetic differences may explain why individuals with similar asbestos exposures develop disease at different rates. Previous studies have frequently reported cases in which individuals develop disease more readily despite being exposed to similar levels of hazardous agents, owing to differences in individual susceptibility [39,40,41,42]. Second, the presence of co-exposures that exacerbate lung damage, such as airborne irritants or non-asbestos dusts commonly found in industrial settings, may influence disease latency. Baur et al. reported that such exposures can lead to airway inflammation and synergistic impairment of lung function, potentially accelerating the onset of asbestosis [22]. In addition, prior studies have shown that air pollution [11], chemical exposures in daily life [43,44,45], and dietary factors [46,47,48] may also contribute to pulmonary vulnerability, highlighting the need for further research into the impact of combined exposures on latency. Lastly, misclassification or recall bias regarding asbestos exposure history may also contribute to variability in latency estimates. This is particularly relevant in cases of environmental exposure, where individuals may be unaware of their initial contact with asbestos, potentially leading to underestimation of the actual latency period due to inaccurate reporting of the first exposure year.

This study has several limitations that should be acknowledged. First, this study is based on a national registry and may be subject to selection and reporting biases, including potential under-ascertainment of mild or asymptomatic cases. Although standardized recognition procedures were used, exposure histories were cross-verified against historical records, and analyses were restricted to asbestosis Grade 1–2, some bias cannot be ruled out. Second, we also did not model interaction terms between smoking and exposure characteristics (e.g., exposure modality or duration) because small subgroup sizes, especially the limited number of current smokers, reduced statistical power and increased the risk of overfitting; accordingly, smoking was incorporated as an adjustment variable. Finally, biomarker data (e.g., genetic susceptibility) were not available, constraining individual-level inference and leaving room for residual confounding. Future work should evaluate interaction effects in larger cohorts and integrate biomarker information through linkage with biobanks or dedicated ancillary studies.

## 5. Conclusions

In this study, we analyzed the latency period of asbestosis in South Korea and the determinants influencing latency. Patients with occupational asbestos exposure had shorter latency periods than those with environmental exposure. Additionally, factors such as type of occupation, distance to the asbestos exposure source, and age at first asbestos exposure were significantly associated with shorter latency periods. These findings highlight the need for further investigation into the characteristics of asbestos exposure to inform the development of national strategies for managing asbestos-related diseases. Given the continued identification of asbestosis cases in Korea, prospective studies on latency periods are warranted.

## Figures and Tables

**Table 1:** Characteristics of study population with asbestosis.

Variables	Asbestosis Grade 1 (Severe)	Asbestosis Grade 2 (Moderate)
	*n* (%)	*n* (%)
Total	234 (100.0)	876 (100.0)
Sex		
Male	198 (84.6)	632 (72.1)
Female	36 (15.4)	244 (27.9)
Age		
<70	29 (12.4)	127 (14.5)
70–79	73 (31.2)	328 (37.4)
80–89	97 (41.5)	335 (38.2)
≥90	35 (15.0)	86 (9.8)
Smoking status		
Never	43 (18.4)	174 (19.9)
Past smoker	22 (9.4)	88 (10.0)
Current smoker	4 (1.7)	10 (1.1)
Unknown	165 (70.5)	604 (68.9)
Exposure modalities		
Occupational	183 (78.2)	577 (65.9)
Environmental	51 (21.8)	299 (34.1)
Diagnosis year		
<2010	7 (3.0)	5 (0.6)
2010–2014	96 (41.0)	289 (33.0)
2015–2019	97 (41.5)	349 (39.8)
≥2020	34 (14.5)	233 (26.6)

**Table 2:** Descriptive statistics on latency period of asbestosis.

Variables	*n*	Mean Latency ^1^ (±SD)	*p*-Value	Median Latency	Range (Min–Max)	5–95 Percentile
Asbestosis (Grade 1)						
Total	234	45.3 (±14.4)		44.0	11.0–83.0	20.0–72.3
Sex						
Male	198	45.1 (±14.5)	0.656	44.0	11.0–83.0	20.0–73.0
Female	36	46.3 (±14.1)		43.5	15.0–78.0	19.3–72.9
Age						
<70	29	30.9 ^a^ (±10.5)	<0.001	33.0	11.0–62.0	14.5–52.5
70–79	73	42.4 ^b^ (±11.6)		43.0	16.0–67.0	19.7–61.3
80–89	97	49.1 ^c^ (±13.2)		49.0	14.0–81.0	28.6–74.2
≥90	35	52.7 ^c^ (±16.1)		46.0	25.0–83.0	31.4–82.2
Asbestosis (Grade 2)						
Total	876	46.3 (±14.9)		45.0	6.0–86.0	20.9–74.2
Sex						
Male	632	45.6 (±13.9)	0.041	45.0	6.0–86.0	22.0–71.0
Female	244	48.1 (±17.0)		46.0	6.0–86.0	20.0–79.0
Age						
<70	127	36.3 ^a^ (±12.5)	<0.001	37.0	6.0–67.0	11.8–58.0
70–79	328	44.3 ^b^ (±12.5)		45.0	6.0–74.0	20.5–68.0
80–89	335	49.9 ^c^ (±15.1)		48.0	11.0–86.0	27.0–77.2
≥90	86	54.6 ^d^ (±15.9)		51.0	23.0–86.0	28.4–84.7

^1^ Same letter indicates statistical insignificance based on Duncan’s multiple comparisons.

**Table 3:** Adjusted ^1^ mean latency periods according to characteristics of the participants.

Variables	Asbestosis (Grade 1)			Asbestosis (Grade 2)		
	*n*	Estimates (95% CI)	*p*-Value	*n*	Estimates (95% CI)	*p*-Value
Sex						
Male	198	44.9 (44.3, 45.6)	0.533	632	46.4 (46.2, 46.7)	0.859
Female	36	44.5 (43.3, 45.7)		244	46.5 (46.1, 46.9)	
Age						
<70	29	30.7 (29.0, 32.3)	<0.001	127	33.7 (33.0, 34.5)	<0.001
70–79	73	40.7 (39.6, 41.9)		328	43.6 (43.1, 44.1)	
80–89	97	49.1 (48.1, 50.2)		335	51.1 (50.7, 51.6)	
≥90	35	56.2 (54.7, 57.8)		86	57.7 (56.8, 58.6)	
Smoking status						
Never	43	45.4 (43.8, 46.9)	0.117	174	47.0 (45.4, 48.5)	0.170
Past smoker	22	45.3 (43.6, 47.0)		88	47.2 (45.5, 48.9)	
Current smoker	4	44.1 (42.0, 46.2)		10	46.5 (44.4, 48.6)	
Unknown	165	44.3 (43.5, 45.1)		604	46.7 (45.9, 47.5)	
Exposure modalities						
Occupational	183	44.4 (43.6, 45.2)	0.010	577	45.0 (44.7, 45.3)	<0.001
Environmental	51	46.0 (44.9, 47.0)		299	47.0 (46.6, 47.3)	
Type of job						
Building ^2^	39	43.8 (42.2, 45.3)	0.214	86	44.5 (43.7, 45.2)	<0.001
Production ^3^	30	42.0 (40.6, 43.5)		90	42.3 (41.8, 42.8)	
Maintenance ^4^	27	43.2 (41.8, 44.5)		94	44.4 (43.7, 45.1)	
Mining ^5^	65	44.1 (42.4, 45.9)		191	45.0 (44.4, 45.6)	
Others	22	44.3 (42.6, 46.1)		116	43.1 (42.5, 43.8)	
Type of exposure source						
Asbestos mines	27	52.5 (49.3, 55.6)	0.348	174	53.8 (52.6, 55.0)	0.001
Asbestos industries	16	47.4 (41.5, 53.3)		76	50.4 (48.8, 52.1)	
Shipyards	4	49.3 (48.1, 50.5)		28	51.1 (50.6, 51.5)	
Asbestos containing building	3	49.0 (45.5, 52.4)		9	51.5 (50.8, 52.2)	
Others	1	49.9 (48.4, 51.4)		12	52.9 (50.9, 54.8)	

^1^ All models were adjusted for sex, age, smoking status, asbestos exposure modalities, and age of first exposure. ^2^ Workers that construct or demolish buildings containing asbestos-containing materials such as insulation. ^3^ Workers that produce asbestos-containing products such as cements, slates, and fabric. ^4^ Workers that maintain and repair asbestos-containing buildings or equipment. ^5^ Workers that extract, convey, and grind asbestos.

**Table 4:** Regression coefficients ^1^ for mean latency periods by continuous exposure indicators after adjusting covariates.

Variables	Asbestosis (Grade 1)		Asbestosis (Grade 2)	
	B (95% CI)	*p*-Value	B (95% CI)	*p*-Value
Occupational exposure				
Exposure duration (years)	−0.051 (−0.109, 0.007)	0.061	−0.159 (−0.288, −0.030)	0.039
Age of first exposure (years)	−0.993 (−1.044, −0.943)	<0.001	−0.952 (−0.977, −0.928)	<0.001
Pack-year	−0.050 (−0.122, 0.022)	0.127	−0.044 (−0.125, 0.037)	0.205
Environmental exposure				
Distance (km)	1.131 (0.001, 2.260)	0.048	0.997 (0.035, 1.959)	0.036
Exposure duration (years)	−0.050 (−0.210, 0.111)	0.971	−0.031 (−0.178, 0.116)	0.710
Age of first exposure (years)	−0.935 (−0.986, −0.884)	<0.001	−0.937 (−0.956, −0.918)	<0.001
Pack-year	−0.053 (−0.119, 0.013)	0.091	−0.047 (−0.122, 0.028)	0.118

^1^ Models on occupational exposure were adjusted for age, exposure duration, age of first exposure, and pack-year. Models on environmental exposure were adjusted for age, distance from the exposure source to the residence, exposure duration, age of first exposure, and pack-year.

## Data Availability

The data presented in this study are available upon request from the corresponding author. The data are not publicly available because they contain sensitive patient information and location data.

## Abbreviations

The following abbreviations are used in this manuscript:

ANOVA	Analysis of variance
ANCOVA	Analysis of covariance

## References

[1] Gulati M., Redlich C.A. (2015). Asbestosis and environmental causes of usual interstitial pneumonia. Curr. Opin. Pulm. Med..

[2] Sporn T.A., Roggli V.L. (2013). Asbestosis. Pathology of Asbestos-Associated Diseases.

[3] Ki Y.-H., Kim J.-M., Roh Y.-M., Chung L., Kim Y.-S., Sim S.-H. (2008). A survey for some asbestos containing products in Korea. J. Environ. Health Sci..

[4] Kang D.-M., Kim J.-E., Lee Y.-J., Lee H.-H., Lee C.-Y., Moon S.-J., Kang M.-S. (2016). Environmental health centers for asbestos and their health impact surveys and activities. Ann. Occup. Environ. Med..

[5] Huh D.-A., Chae W.-R., Choi Y.-H., Kang M.-S., Lee Y.-J., Moon K.-W. (2022). Disease latency according to asbestos exposure characteristics among malignant mesothelioma and asbestos-related lung cancer cases in South Korea. Int. J. Environ. Res. Public Health.

[6] Guerreschi E., Dragoni L., Sisinni A., Fabrizi S., Miceli G., Nante N. (2024). Asbestos is still paid for dearly. Eur. J. Public Health.

[7] Marinaccio A., Binazzi A., Cauzillo G., Cavone D., De Zotti R., Ferrante P., Gennaro V., Gorini G., Menegozzo M., Mensi C. (2007). Analysis of latency time and its determinants in asbestos related malignant mesothelioma cases of the Italian register. Eur. J. Cancer.

[8] Magnani C., Ferrante D., Barone-Adesi F., Bertolotti M., Todesco A., Mirabelli D., Terracini B. (2008). Cancer risk after cessation of asbestos exposure: A cohort study of Italian asbestos cement workers. Occup. Environ. Med..

[9] Baur X. (2018). Asbestos-related disorders in Germany: Background, politics, incidence, diagnostics and compensation. Int. J. Environ. Res. Public Health.

[10] Petrof O., Neyens T., Nuyts V., Nackaerts K., Nemery B., Faes C. (2020). On the impact of residential history in the spatial analysis of diseases with a long latency period: A study of mesothelioma in Belgium. Stat. Med..

[11] Huh D.-A., Choi Y.-H., Kim L., Park K., Lee J., Hwang S.H., Moon K.W., Kang M.-S., Lee Y.-J. (2024). Air pollution and survival in patients with malignant mesothelioma and asbestos-related lung cancer: A follow-up study of 1591 patients in South Korea. Environ. Health.

[12] Kim S.-Y., Kim Y.-C., Kim Y., Hong W.-H. (2016). Predicting the mortality from asbestos-related diseases based on the amount of asbestos used and the effects of slate buildings in Korea. Sci. Total Environ..

[13] Kwak K.M., Paek D., Hwang S.-S., Ju Y.-S. (2017). Estimated future incidence of malignant mesothelioma in South Korea: Projection from 2014 to 2033. PLoS ONE.

[14] Kwak K., Cho S.-I., Paek D. (2021). Future incidence of malignant mesothelioma in South Korea: Updated projection to 2038. Int. J. Environ. Res. Public Health.

[15] Frost G. (2013). The latency period of mesothelioma among a cohort of British asbestos workers (1978–2005). Br. J. Cancer.

[16] Nielsen L.S., Baelum J., Rasmussen J., Dahl S., Olsen K.E., Albin M., Hansen N.C., Sherson D. (2014). Occupational asbestos exposure and lung cancer—A systematic review of the literature. Arch. Environ. Occup. Health.

[17] Lanphear B.P., Buncher C.R. (1992). Latent period for malignant mesothelioma of occupational origin. J. Occup. Med..

[18] Fisher S.A., Patrick K., Hoang T., Marcq E., Behrouzfar K., Young S., Miller T.J., Robinson B.W.S., Bueno R., Nowak A.K. (2024). The MexTAg collaborative cross: Host genetics affects asbestos related disease latency, but has little influence once tumours develop. Front. Toxicol..

[19] Ferri G.M., Guastadisegno C.M., Intranuovo G., Luisi V., Cavone D., Licchelli B., Buononato E.V., Macinagrossa L., Molinini R. (2016). Relationship between the Asbestos Cumulative Exposure Index (ACEI) and the Latency Period of Asbestos Related Diseases (ARD) within an Italian Study Group of Ex-Asbestos Workers. Occup. Med. Health Aff..

[20] Dominguez C.E., González M.M., Molina A.H., Ramírez I.M., Andreu A.M., Valero F.R., Doña J.A.C., Jiménez A.L. (2020). Characteristics of Patients Evaluated in Specialized Post-Asbestos Exposure Outpatient Clinic. Eur. Respir. Soc..

[21] Lazarus A., Massoumi A., Hostler J., Hostler D.C. (2012). Asbestos–Related Pleuropulmonary Diseases: Benign and Malignant. Postgrad. Med..

[22] Baur X., Manuwald U., Wilken D. (2010). Does long-term asbestos exposure cause an obstructive ventilation pattern?. Pneumologie.

[23] Henderson I., Sterman D.H., Smith R.L., Rom W.N. (2022). Asbestosis. Modern Occupational Diseases: Diagnosis, Epidemiology, Management and Prevention.

[24] Liddell D. (2024). Asbestos in the occupational environment. Mineral Fibers and Health.

[25] Kurunthachalam S. (2013). Asbestos and Its Toxicological Concern. Hydrol. Curr. Res..

[26] Brody A.R., Laurent G.J., Shapiro S.D. (2006). OCCUPATIONAL DISEASES | Asbestos-Related Lung Disease. Encyclopedia of Respiratory Medicine.

[27] Larrouy C., Tandjaoui-Lambiotte H., Mellat M., Fabre C., Defrejacques C., Adotti F., Piquet J. (1990). Environmental interstitial pneumonia caused by asbestos. Study of a Turkish family exposed to tremolite. Rev. Pneumol. Clin..

[28] Luo S., Liu X., Mu S., Tsai S., Wen C. (2003). Asbestos related diseases from environmental exposure to crocidolite in Da-yao, China. I. Review of exposure and epidemiological data. Occup. Environ. Med..

[29] Banks D.E., Morris M.J., Jindal S.K. (2014). Asbestos Fibers: Mechanisms of Injury. Studies on Respiratory Disorders.

[30] Popper H. (2016). Pneumoconiosis and Environmentally Induced Lung Diseases. Pathology of Lung Disease: Morphology–Pathogenesis–Etiology.

[31] Ministry of Employment and Labor (MOEL) (1984). Investigation on the Actual Condition of Hazardous Environment at Asbestos Handling Workplaces.

[32] Kim S. (2019). Clinical Characteristics and Long-Term Follow-Up of Asbestos in Workers at One Asbestos Textile Factory in Busan.

[33] Choi J.K., Paek D.M., Paik N.W., Hisanaga N., Sakai K. (1998). A study on several minerals contaminated with asbestiform fibers in Korea. J. Korean Soc. Occup. Environ. Hyg..

[34] Smith C.M., Kelsey K.T., Wiencke J.K., Leyden K., Levin S., Christiani D.C. (1994). Inherited glutathione-S-transferase deficiency is a risk factor for pulmonary asbestosis. Cancer Epidemiol. Biomark. Prev. Publ. Am. Assoc. Cancer Res. Cosponsored Am. Soc. Prev. Oncol..

[35] Kukkonen M.K., Hämäläinen S., Kaleva S., Vehmas T., Huuskonen M.S., Oksa P., Vainio H., Piirilä P., Hirvonen A. (2011). Genetic susceptibility to asbestos-related fibrotic pleuropulmonary changes. Eur. Respir. J..

[36] Franko A., Dolžan V., Arneric N., Dodic-Fikfak M. (2008). The influence of genetic polymorphisms of GSTP1 on the development of asbestosis. J. Occup. Environ. Med..

[37] Kuzmina L.P., Khotuleva A.G., Kovalevsky E.V., Anokhin N.N., Tskhomariya I.M., Хoтулева А. (2020). Association of genetic polymorphism of cytokines and antioxidant enzymes with the development of asbestosis. Meditsina Tr. I Promyshlennaya Ekol..

[38] Kukkonen M.K., Vehmas T., Piirilä P., Hirvonen A. (2014). Genes involved in innate immunity associated with asbestos-related fibrotic changes. Occup. Environ. Med..

[39] Choi Y.-H., Kim L., Huh D.-A., Moon K.W., Kang M.-S., Lee Y.-J. (2024). Association between oil spill clean-up work and thyroid cancer: Nine years of follow-up after the Hebei Spirit oil spill accident. Mar. Pollut. Bull..

[40] Hwang S.H., Lee Y.-J., Choi Y.-H., Huh D.-A., Kang M.-S., Moon K.W. (2024). Long-term effects of the Hebei Spirit oil spill on the prevalence and incidence of allergic disorders. Sci. Total Environ..

[41] Kim L., Huh D.-A., Kang M.-S., Park K., Lee J., Hwang S.H., Choi H.J., Lim W., Moon K.W., Lee Y.-J. (2024). Chemical exposure from the Hebei spirit oil spill accident and its long-term effects on mental health. Ecotoxicol. Environ. Saf..

[42] Lee J., Huh D.-A., Kim L., Park K., Choi Y.-H., Hwang S.-H., Lim W., Choi H.J., Moon K.W., Kang M.-S. (2025). The short-term and long-term effects of oil spill exposure on dyslipidemia: A prospective cohort study of Health Effects Research on the Hebei Spirit Oil Spill. Mar. Pollut. Bull..

[43] Choi Y.-H., Huh D.-A., Kim L., ji Lee S., Moon K.W. (2024). Health risks of pest control and disinfection workers after the COVID-19 outbreak in South Korea. J. Environ. Sci..

[44] Hwang S.-H., Choi Y.-H., Huh D.-A., Kim L., Park K., Lee J., Choi H.J., Lim W., Moon K.W. (2025). Per-and polyfluoroalkyl substances exposures are associated with non-alcoholic fatty liver disease, particularly fibrosis. Environ. Pollut..

[45] Park K., Huh D.-A., Kim L., Choi Y.-H., Lee J., Hwang S.H., Choi H.J., Lim W., Moon K.W. (2025). Levels of serum per-and polyfluoroalkyl substances and association with dyslipidemia in the Korean population. Ecotoxicol. Environ. Saf..

[46] Zheng P.-F., Shu L., Si C.-J., Zhang X.-Y., Yu X.-L., Gao W. (2016). Dietary patterns and chronic obstructive pulmonary disease: A meta-analysis. COPD J. Chronic Obstr. Pulm. Dis..

[47] Kim L., Choi Y.-H., Huh D.-A., Moon K.W. (2024). Associations of minimally processed and ultra-processed food intakes with cardiovascular health in Korean adults: The Korea National Health and Nutrition Examination Survey (KNHANES VI), 2013–2015. J. Expo. Sci. Environ. Epidemiol..

[48] Kim L., Huh D.-A., Park K., Lee J., Hwang S.-H., Choi H.J., Lim W., Moon K.W. (2025). Dietary exposure to environmental phenols and phthalates in Korean adults: Data analysis of the Korean National Environmental Health Survey (KoNEHS) 2018–2020. Int. J. Hyg. Environ. Health.

